# Esthetic Crown Lengthening: A Case Report and Literature Review

**DOI:** 10.7759/cureus.113868

**Published:** 2026-08-03

**Authors:** Zineb Essadeq, Bouchra Elhouari, Jamila Kissa

**Affiliations:** 1 Periodontology, Hassan II University of Casablanca, Casablanca, MAR

**Keywords:** altered passive eruption, crown lengthening surgery, gingival aesthetics, gummy smile, gummy smile correction

## Abstract

Excessive gingival display is a common esthetic concern among patients seeking smile enhancement. Altered passive eruption (APE) is a frequent etiologic factor and is characterized by incomplete apical migration of the gingival margin following tooth eruption. Accurate diagnosis and adherence to periodontal biologic principles are essential for achieving stable and predictable outcomes in esthetic crown lengthening (ECL). This article reviews the biologic and surgical principles of ECL, with particular emphasis on APE, and illustrates its management through a clinical case. A 28-year-old systemically healthy woman presented with short clinical crowns and excessive gingival display in the maxillary anterior region. Clinical and radiographic examination revealed the alveolar crest at or near the cemento-enamel junction (CEJ), consistent with APE Type I, Subgroup B (Type I-B) according to Coslet's classification. ECL was performed using a full-thickness flap with osseous recontouring to re-establish the supracrestal tissue attachment and achieve harmonious dentogingival proportions. Healing was uneventful, and stable gingival margins were maintained at the six-month follow-up, with marked improvement in smile esthetics. ECL is a predictable and effective treatment for APE when appropriate diagnosis, meticulous surgical planning, and preservation of the supracrestal tissue attachment are respected. In Type I-B cases, osseous resection is necessary to ensure long-term periodontal stability and optimal esthetic outcomes.

## Introduction

A harmonious smile is a fundamental component of facial esthetics and plays a significant role in social interactions and self-esteem. Among patients seeking esthetic dental treatment, excessive gingival display, commonly referred to as a "gummy smile," is a frequent concern. This condition may result from various etiologies, including altered passive eruption (APE), gingival overgrowth, vertical maxillary excess, or a short or hypermobile upper lip.

Clinical crown lengthening is a surgical procedure performed to expose additional tooth structure for restorative or esthetic purposes by apically repositioning the gingival margin. Depending on the underlying anatomical and biologic conditions, the procedure may be accomplished through gingivectomy or an apically positioned flap, with or without osseous resection [1,2].

Accurate diagnosis and individualized treatment planning are essential to preserve the supracrestal tissue attachment and ensure long-term stability of the gingival margin while achieving optimal esthetic outcomes. When appropriately indicated and meticulously executed, esthetic crown lengthening (ECL) is a predictable procedure that can significantly improve smile harmony without compromising periodontal health.

The aim of this article is to review the biologic and surgical principles of ECL, with particular emphasis on APE, and to illustrate its clinical application through a case report.

## Case presentation

Diagnosis and planning

**Figure 1:**
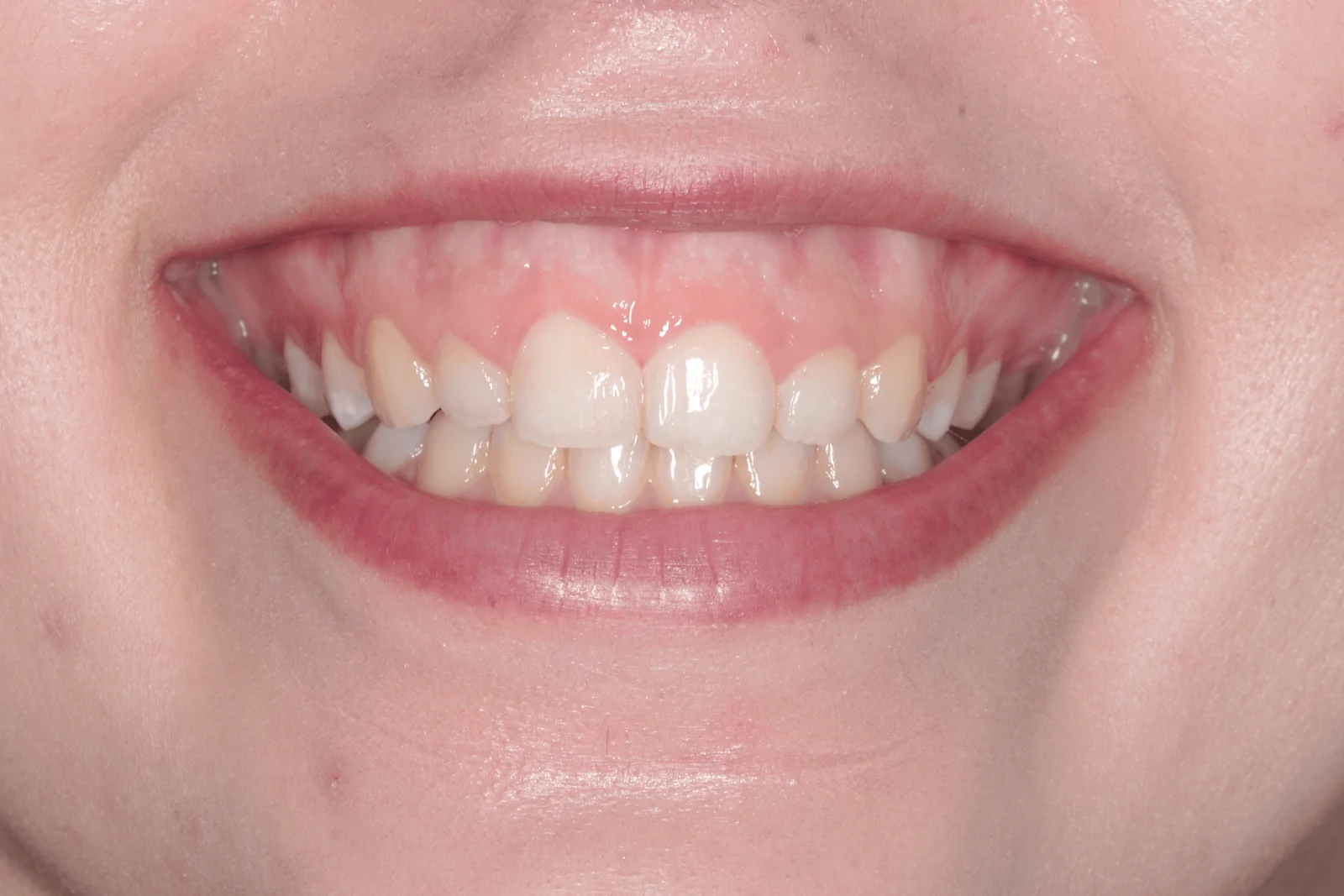
Preoperative frontal smile view showing excessive gingival display in the maxillary anterior region.

A 28-year-old systemically healthy woman presented with the chief complaint of dissatisfaction with the esthetic appearance of her smile. She had previously undergone orthodontic treatment, which was successfully completed but failed to resolve her primary esthetic concerns, namely excessive gingival display (gummy smile) and the appearance of short maxillary anterior teeth (Figure 1).

**Figure 2:**
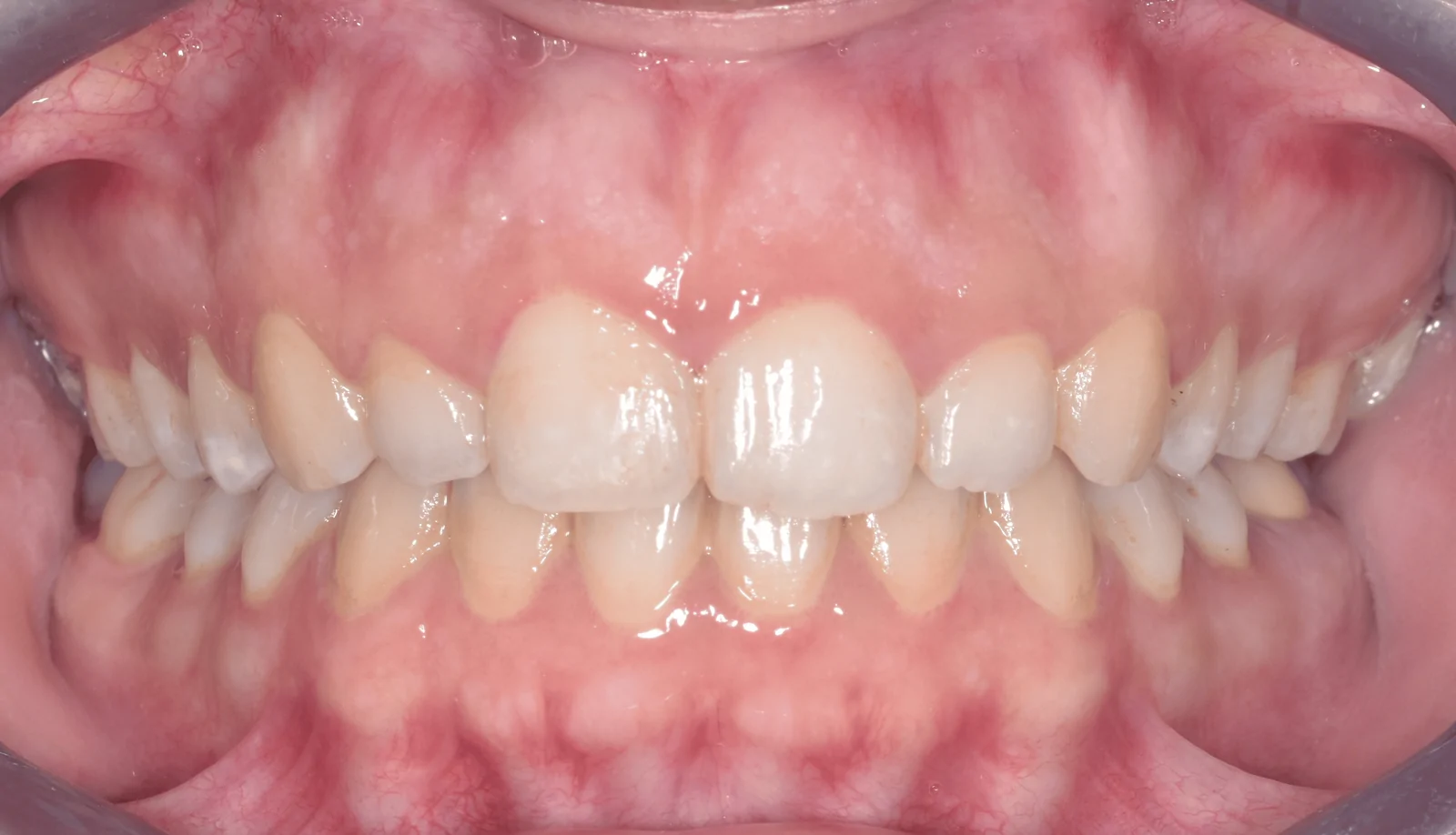
Preoperative intraoral frontal view illustrating short clinical crowns and altered dentogingival proportions.

Clinical examination revealed a short upper lip and excessive gingival coverage of the maxillary anterior teeth. The probing depth was 1 mm around the treated teeth, and the width of keratinized tissue ranged from 5 to 7 mm. The gingival margins were positioned coronally, resulting in short clinical crowns consistent with APE. The patient expressed interest in improving her smile through periodontal esthetic intervention (Figure 2).

**Figure 3:**
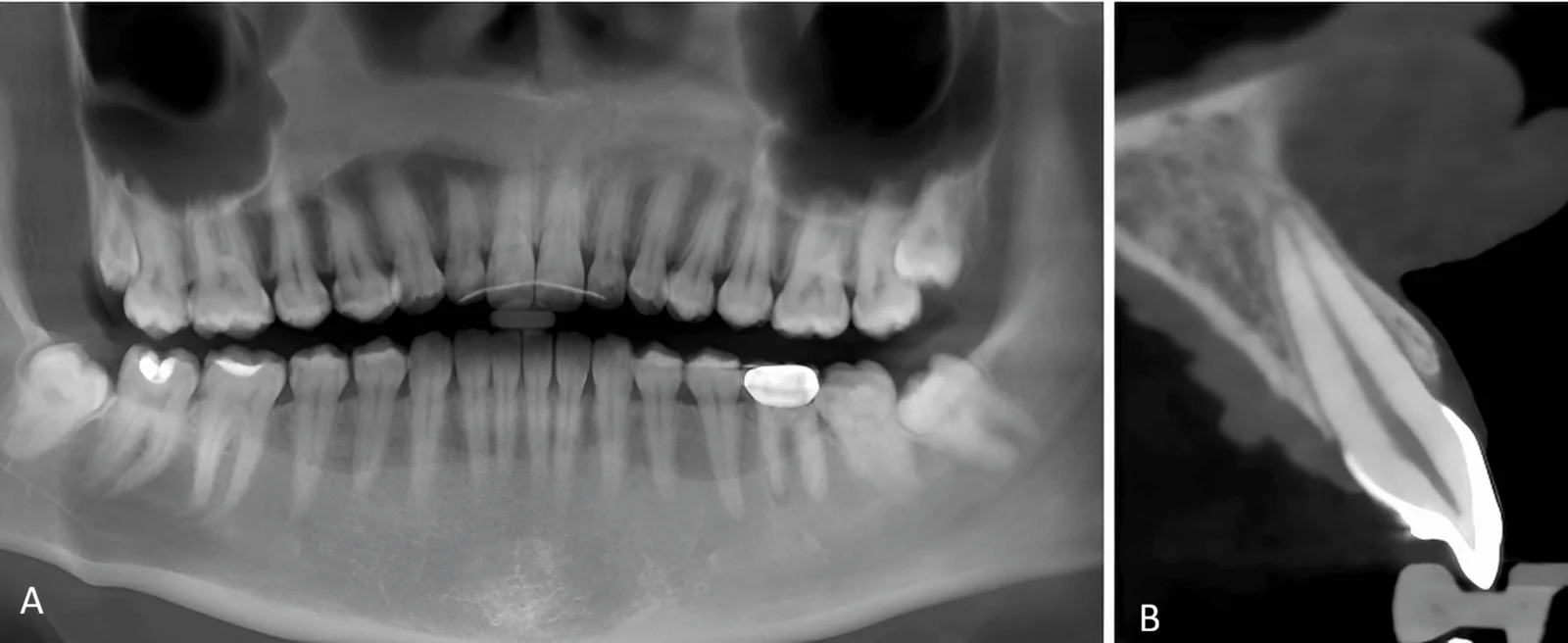
Preoperative radiographic assessment of altered passive eruption (APE) Type I-B. (A) Panoramic radiograph showing the alveolar crest positioned at or near the cemento-enamel junction (CEJ), consistent with APE Type I-B. (B) Cone-beam computed tomography (CBCT) sagittal section confirming the close proximity of the alveolar crest to the CEJ.

Cone-beam computed tomography (CBCT) was performed to evaluate the relationship between the cemento-enamel junction (CEJ) and the alveolar crest (Figure 3). The clinical and radiographic findings were consistent with APE Type I, Subgroup B (Type I-B), characterized by excessive gingival coverage of the anatomical crowns and an alveolar crest located close to the CEJ. Based on these diagnostic criteria, ECL using an apically positioned flap combined with osseous recontouring was selected to re-establish an adequate supracrestal tissue attachment and achieve optimal esthetic and functional outcomes.

The ECL procedure involved the maxillary teeth #16 to #25. Clinical examination revealed short clinical crowns with excessive gingival display. Periodontal probing depth was 1 mm, and the width of keratinized tissue ranged from 5 to 7 mm. CBCT evaluation demonstrated that the alveolar crest was located close to the CEJ, consistent with APE Type I-B. These clinical and radiographic findings constituted the diagnostic basis for selecting ECL using an apically positioned flap with osseous recontouring.

Surgical procedure

**Figure 4:**
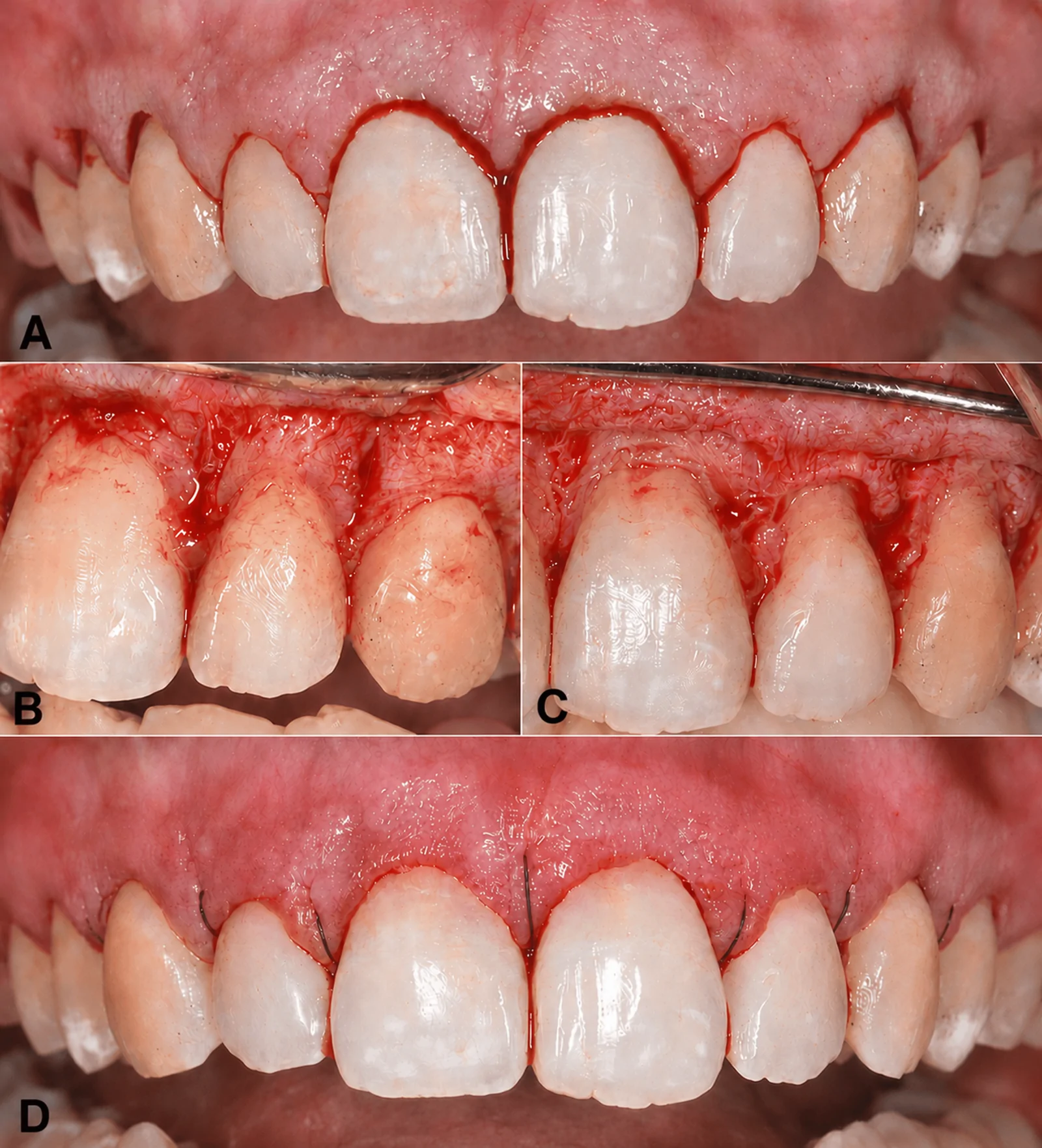
Surgical markings outlining the planned new gingival margin according to ideal esthetic proportions. (A) Internal bevel incision before flap elevation. (B) Full-thickness flap elevation exposing the underlying alveolar bone. (C) Intraoperative view following osteotomy and osteoplasty to re-establish the supracrestal tissue attachment. (D) Flap repositioned and secured with uninterrupted sutures.

Following the clinical and radiographic diagnosis of APE, we planned a surgical crown lengthening procedure with limited flap elevation and osseous recontouring to ensure long-term stability of the gingival margins and to preserve the biologic width. After administering local anesthesia, the surgical procedure began with an internal bevel gingivectomy from teeth #16 to #25 to remove the excessive gingival tissue and to restore natural tooth proportions. Then, a full-thickness mucoperiosteal flap was carefully reflected from teeth #16 to #25. We performed a selective ostectomy and osteoplasty to create at least 3 mm of space between the new gingival margin and the alveolar crest. We repositioned and sutured the flap (Figure 4). The procedure was completed with minimal trauma, and the patient was given postoperative instructions, including analgesics and chlorhexidine rinses, along with a follow-up schedule to monitor healing and tissue response. Postoperative care included a soft diet for the first few days and avoiding hot or spicy foods. Sutures were scheduled for removal after eight days. Routine follow-up visits were arranged at one week, two weeks, one month, three months, and one year.

**Figure 5:**
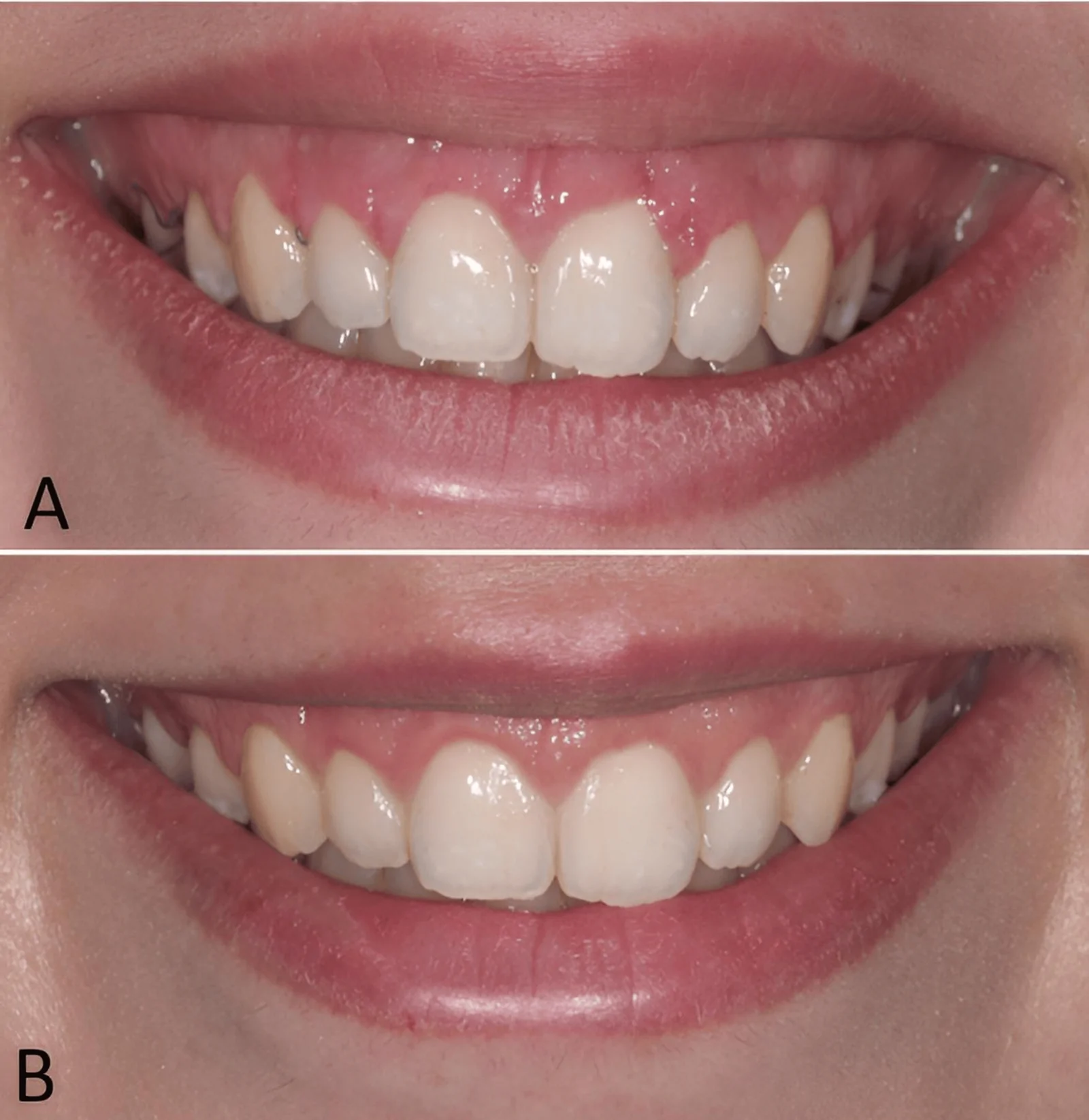
(A) Early postoperative view (eight days) during suture removal. (B) One-year postoperative smile view showing harmonious gingival architecture and enhanced esthetics.

After one week of healing, mild papillary inflammation was observed, and the sutures were removed without incident. After four weeks, the gingival tissues showed healthy pink coloration and proper adaptation to the underlying bone with a stabilized gingival margin. At this stage, the clinical crown length appeared significantly improved. At one year, the final gingival contours were well established, and the esthetic outcome was highly satisfactory (Figure 5). The patient expressed a high level of satisfaction with the appearance of her smile, reporting increased confidence and a positive emotional impact. No relapse of gingival tissue or inflammation was observed during the long-term follow-up period.

## Discussion

APE: diagnosis and classification

APE is a developmental condition characterized by incomplete apical migration of the gingival margin to the CEJ after the tooth has reached its functional position. Clinically, APE manifests as short clinical crowns and excessive gingival display, often leading to esthetic concerns despite normal tooth morphology [3]. 

Management requires careful evaluation of the supracrestal tissue attachment (historically termed “biologic width”), which comprises the junctional epithelium (mean ≈ 0.97 mm) and the supracrestal connective tissue attachment (mean ≈ 1.07 mm), summing to approximately 2 mm (Gargiulo et al., 1961; Ingber et al., 1977) [4,5].

In some cases, osseous resection may be necessary to establish adequate biologic dimensions. Surgical correction aims not only to restore esthetic proportions but also to achieve a stable, predictable gingival margin while minimizing the risk of recession or tissue rebound (Coslet et al., 1977; Mele et al., 2018; Zucchelli and Mounssif, 2015) [6-8].

Diagnosis

The diagnosis of APE requires a comprehensive evaluation that integrates clinical, radiographic, and periodontal assessments. Key diagnostic steps include: 

Clinical examination: Measurement of the gingival margin relative to the CEJ, probing depth, and width of keratinized tissue. Careful analysis of tooth proportions and smile line helps to quantify the esthetic impact [7,9].

Bone sounding: Under local anesthesia, bone sounding allows direct assessment of the supracrestal tissue attachment and helps distinguish APE from gingival overgrowth or hyperplastic conditions [6].

Radiographic assessment: Periapical or bitewing radiographs are useful for evaluating the position of the alveolar bone crest relative to the CEJ, providing valuable information for surgical planning [6]. However, these conventional radiographs have limitations in accurately assessing the facial alveolar bone level. Therefore, CBCT may be indicated in selected cases to confirm transgingival bone sounding findings and provide a more precise three-dimensional evaluation of the osseous architecture.

Accurate differentiation from other causes of excessive gingival display, such as vertical maxillary excess, hypermobile upper lip, or gingival enlargement, is essential to avoid inappropriate treatment.

Classification

The most widely accepted classification of APE was proposed by Coslet et al. (1977) [6]. This system distinguishes two main types according to the width of keratinized gingiva and the position of the mucogingival junction, with each type further subdivided based on the level of the alveolar bone relative to the CEJ:

Type I: High width of keratinized gingiva.

Type II: Normal width of keratinized gingiva.

Subgroup A: Alveolar bone crest is positioned 2-3 mm apical to the CEJ (physiologic).

Subgroup B: Alveolar bone crest is located at or near the CEJ (coronal), requiring osseous resection.

This classification is clinically valuable, as it directly informs the surgical approach. In Type I-A or II-A cases, soft tissue repositioning alone may be sufficient, whereas Subgroup B cases typically necessitate combined gingival and osseous surgery to restore an appropriate supracrestal tissue attachment and establish long-term gingival stability.

Esthetic crown lengthening

Crown lengthening can be broadly classified as either functional or esthetic, depending on the primary clinical objective. While functional crown lengthening aims to expose subgingival tooth structure for restorative purposes with restoration of the biologic width, as originally described by Gargiulo et al. (1961) [4], ECL focuses on optimizing gingival symmetry, proportion, and harmony to improve overall facial and dental esthetics. Modern approaches often combine these objectives to address both esthetic and functional requirements in the anterior maxilla.

Critical Considerations for Esthetic Crown Lengthening

Although the Coslet classification remains a cornerstone, recent studies emphasize the importance of individualized treatment planning. Factors such as periodontal phenotype, gingival thickness, and patient-specific esthetic expectations can influence both the choice of surgical technique and postoperative outcomes [8,10]. Personalized approaches that integrate these variables have been associated with improved stability of the gingival margin, reduced risk of relapse, and enhanced patient satisfaction. Multiple surgical techniques are available to achieve ECL, and the choice of approach is guided by clinical and radiographic evaluation, as described previously. A key consideration in any crown lengthening procedure is the preservation of the supracrestal tissue attachment, approximately 2-3 mm. Respecting this dimension is essential for maintaining periodontal health and ensuring long-term stability of surgical outcomes [4,5].

Clinical attachment level (CAL) is a critical parameter for assessing the effectiveness of ECL. While the most pronounced tissue changes occur at surgically treated sites, adjacent or non-adjacent teeth may also demonstrate soft tissue alterations, potentially affecting esthetic outcomes, particularly in the anterior zone. Factors such as periodontal phenotype, tooth morphology, surgical technique, and healing duration significantly influence final outcomes and should be carefully considered during treatment planning [10].

Emerging techniques

Minimally invasive flapless (FL) approaches, often using piezoelectric instruments, have been proposed as alternatives to traditional open-flap (OF) techniques in selected esthetic cases. Evidence suggests that FL procedures may offer improved gingival margin stability, reduced postoperative discomfort, and higher patient satisfaction, particularly in cases of APE [11,12]. Nonetheless, OF approaches remain necessary when extensive osseous reduction or enhanced visibility is required.

Radiographic evaluation supports these findings, demonstrating that well-executed crown lengthening promotes favorable bone remodeling, functional stability, and esthetic improvement in anterior maxillary restorations [13]. Successful outcomes depend on comprehensive clinical and radiographic assessments, individualized surgical planning, and strict adherence to periodontal principles to minimize complications such as delayed soft tissue healing, sensitivity, or esthetic relapse [10,14,15].

Outcomes and predictability

Systematic reviews and clinical studies have demonstrated that ECL generally results in stable periodontal parameters and predictable esthetic outcomes when biologic width is preserved. Pontoriero and Carnevale (2001) [10] and Camargo et al. (2007) [14] reported that respecting the biologic width leads to stable gingival margins and favorable esthetic results. Similarly, Al-Sowygh (2019) [15] observed significant improvements in CAL and probing depth, as well as stable gingival margins, confirming the predictability of surgical crown lengthening in increasing clinical crown height without compromising esthetics.

Despite predictable outcomes, some variability exists. Soft tissue rebound or apical migration of the gingival margin can occur, particularly in cases lacking standardized postoperative care or in patients with thin periodontal biotypes. These observations underscore the importance of adequate healing time, careful postoperative monitoring, and individualized surgical planning to minimize complications such as gingival recession or esthetic relapse [7,13,15].

Recent advances in ECL have incorporated digital workflows, including Digital Smile Design (DSD), intraoral scanning, CBCT, and computer-guided surgical templates, allowing more accurate diagnosis, treatment planning, and predictable esthetic outcomes. Furthermore, the current concept of supracrestal tissue attachment (STA), introduced by the 2017 World Workshop, has replaced the traditional term "biologic width" and emphasizes the importance of preserving periodontal tissue dimensions to ensure long-term periodontal health and restorative success [16].

Clinical implications of the present case

In the present case, radiographic evaluation confirmed the need for osseous recontouring. A full-thickness flap approach allowed direct visualization of the bone crest and precise osteotomy, ensuring adequate re-establishment of the biologic dimension.

Healing dynamics following ECL are influenced by several factors, including periodontal biotype, flap design, and the extent of osseous resection. Thick biotypes, such as in the present case, are generally associated with greater tissue stability. A tendency for coronal rebound can be observed if adequate bone reduction is not achieved. No gingival relapse was observed at the six-month follow-up, suggesting that the biologic principles were respected and sufficient supracrestal space was created.

The stability observed in this case is consistent with the existing literature indicating that proper surgical planning and adherence to periodontal biologic principles lead to predictable and stable outcomes in APE management.

Although minimally invasive FL techniques have been proposed in selected cases, OF approaches remain the gold standard in Type I-B APE due to the necessity of precise osseous modification.

The present case is consistent with previously published reports demonstrating that ECL with an apically positioned flap and osseous recontouring is a predictable approach for the management of APE Type I-B. Similar to earlier studies, our patient achieved harmonious gingival contours, improved crown proportions, and satisfactory esthetic outcomes while maintaining periodontal health. Unlike reports using DSD or computer-guided surgical templates, the present case was managed using a conventional surgical approach supported by thorough clinical examination and CBCT evaluation. Although digital workflows may enhance treatment planning and precision, the favorable clinical outcome observed in this case suggests that conventional techniques remain effective when appropriate diagnosis and biological principles are respected. Nevertheless, longer follow-up periods are desirable to further confirm the long-term stability of the periodontal and esthetic outcomes [17].

## Conclusions

Crown lengthening is a reliable and versatile technique that, when carefully planned and executed, allows optimal periodontal, functional, and esthetic outcomes. Minimally invasive approaches appear particularly advantageous in suitable cases. However, the long-term success of ECL relies on respecting biologic principles, individualized treatment planning, and proper postoperative management. Further well-designed studies with long-term follow-up are still needed to refine clinical protocols and enhance predictability across different patient profiles.
